# The Human Health Implications of Antibiotic Resistance in Environmental Isolates from Two Nebraska Watersheds

**DOI:** 10.1128/spectrum.02082-21

**Published:** 2022-03-21

**Authors:** Linsey Donner, Zachery R. Staley, Jonathan Petali, Jodi Sangster, Xu Li, Wayne Mathews, Daniel Snow, Adina Howe, Michelle Soupir, Shannon Bartelt-Hunt

**Affiliations:** a College of Allied Health Professions, University of Nebraska Medical Center, Omaha, Nebraska, USA; b Department of Civil and Environmental Engineering, University of Nebraska – Lincoln, Lincoln, Nebraska, USA; c Environmental Health Program, New Hampshire Department of Environmental Services, Concord, New Hampshire, USA; d Nebraska Water Center, University of Nebraska-Lincoln, Lincoln, Nebraska, USA; e Department of Agricultural and Biosystems Engineering, Iowa State University, Ames, Iowa, USA; University of Nebraska-Lincoln

**Keywords:** antibiotic resistance, antibiotics, watersheds

## Abstract

One Health field-based approaches are needed to connect the occurrence of antibiotics present in the environment with the presence of antibiotic resistance genes (ARGs) in Gram-negative bacteria that confer resistance to antibiotics important in for both veterinary and human health. Water samples from two Nebraska watersheds influenced by wastewater effluent and agricultural runoff were tested for the presence of antibiotics used in veterinary and human medicine. The water samples were also cultured to identify the bacteria present. Of those bacteria isolated, the Gram-negative rods capable of causing human infections had antimicrobial susceptibility testing and whole-genome sequencing (WGS) performed to identify ARGs present. Of the 211 bacterial isolates identified, 37 belonged to pathogenic genera known to cause human infections. Genes conferring resistance to beta-lactams, aminoglycosides, fosfomycins, and quinolones were the most frequently detected ARGs associated with horizontal gene transfer (HGT) in the watersheds. WGS also suggest recent HGT events involving ARGs transferred between watershed isolates and bacteria of human and animal origins. The results of this study demonstrate the linkage of antibiotics and bacterial ARGs present in the environment with potential human and/or veterinary health impacts.

**IMPORTANCE** One health is a transdisciplinary approach to achieve optimal health for humans, animals, plants and their shared environment, recognizing the interconnected nature of health in these domains. Field based research is needed to connect the occurrence of antibiotics used in veterinary medicine and human health with the presence of antibiotic resistance genes (ARGs). In this study, the presence of antibiotics, bacteria and ARGs was determined in two watersheds in Nebraska, one with agricultural inputs and the other with both agricultural and wastewater inputs. The results presented in this study provide evidence of transfer of highly mobile ARG between environment, clinical, and animal-associated bacteria.

The spread of antibiotic resistance is considered to be a significant global health challenge (1). One primary cause of the spread of antibiotic resistance is overuse in both clinical and agricultural settings. In the United States., nearly 300 million antibiotic prescriptions are given annually, with an estimate that 30% of those prescriptions were not necessary (2). Antibiotics can be excreted unmetabolized and enter wastewater treatment plants, where they are typically detected in treated wastewater effluent (3). Antibiotic use in livestock operations has been estimated to account for as much as 80% of the total antibiotic consumption in the United States. (4) for disease treatment and prevention (5, 6). Manure and wastewater from livestock operations has been shown to contain antibiotics and antibiotic resistant bacteria, and application of this material to cropland as a soil fertilizer can contribute to agricultural runoff to adjacent surface waters, introducing antibiotics and antibiotic resistant bacteria into the environment (7). These clinical and agricultural practices can proliferate antibiotic resistant bacteria, with discharge/runoff from these anthropogenic sources entering the environment and potentially impacting autochthonous bacteria, increasing antibiotic resistance in the environment, and negatively impacting human and animal health (8).

The presence of antibiotic resistance genes (ARGs) can pose a risk to public health even when these genes occur in commensal bacteria. ARGs can be transferred between bacteria as a result of horizontal gene transfer via mobile genetic elements (MGE; such as plasmids, gene cassettes, transposons and integrons) (9–11). For example, one study has demonstrated the capacity for Enterobacter spp. to act as a reservoir of extended-spectrum β-lactamase-producing (ESBL) genes that may be transferred to Escherichia coli (12). The danger of mobile ARGs (i.e., ARGs associated with an MGE) being transferred to clinically relevant pathogens increases under selective pressure, such as in the effluent or runoff from hospitals, wastewater treatment plants, livestock operations or agricultural fields fertilized with manure (3, 9, 13, 14). Consequently, to obtain a better understanding of the spread of ARGs in the environment, it is necessary to look at interconnections between humans, animals and the environment.

The One Health perspective is a concept that focuses on linkages between humans, animals, and the environment, particularly focusing on the spread of antibiotic resistance. The Centers for Disease Control and Prevention (CDC) has identified several antibiotic resistant pathogens to be of primary concern, including Salmonella, Campylobacter jejuni, *Clostridioides (Clostridium) difficile*, methicillin-resistant Staphylococcus aureus (MRSA), and particularly extended spectrum beta-lactamases (ESBL) *Enterobacteriaceae* (15). Several studies have demonstrated connections of these pathogens in humans, animals, and the environment. For example, a study of source and geographic differences of Salmonella enterica serovar Newport found genetic similarities in ARGs between strains found in humans and animals (16). Another study examining S. enterica noted ARG sequence homology between clinical and bovine samples in Canada (17). Klebsiella pneumoniae isolated from retail meats was found to have homologous ARG sequences to human clinical isolates from urinary tract infections (18). K. pneumoniae isolates obtained from hospitals were also found to have similar ARG sequences to environmental isolates found in surface waters adjacent to hospital wastewater discharges (19). These studies highlight the interconnectedness of humans, animals, and the environment and demonstrate the necessity of utilizing a One Health approach to better understand the transmission of antibiotic resistance.

While numerous studies have examined antibiotic resistance from foodborne illness at the interface of human and animal health, fewer studies have investigated environmental links to human pathology and antibiotic resistance. The widespread usage of antibiotics in livestock operations and occurrence of antibiotics in wastewater discharges results in the potential for these sources to contribute novel ARGs into the environment which may have clinical impacts, necessitating further study. In this study, a One Health approach was utilized to characterize the concentration of antibiotics and antibiotic resistance profiles of Gram-negative bacteria in two Nebraska watersheds. The first watershed is influenced by wastewater effluent and agricultural runoff and the second is primarily influenced by agricultural runoff. Whole genome sequencing was conducted on the bacteria known to cause human infections to search for ARGs that have previously been characterized as acquired via horizontal gene transfer (HGT) between different genera and to identify potential linkages between the watershed isolates and bacteria of human and animal origins.

## RESULTS AND DISCUSSION

### Antibiotic occurrence.

Of the veterinary and clinical antibiotics targeted for analysis, 31 were detected during this study (Table S3). Monensin was found in all samples at concentrations ranging between 3.4 to 718.7 ng POCIS^−1^. Lincomycin (95.8%) and tylosin (79.2%) were frequently detected in samples with ranges of 1.13–163.6 and 0.10–134.76 ng POCIS^−1^, respectively (Table S3). Sulfathiazole, sulfanilamide, oxytetracycline, tetracycline, and sulfadiazine were only found in the Elkhorn River watershed which was influenced by both animal production facilities as well as wastewater treatment effluent; while sulfachlorpyridazine, clinafloxacin, and penicilin G were only detected within the Shell Creek watershed which was influenced by animal production facilities with minimal municipal wastewater inputs. Ceftiofur, novobiocin, penillic acid, virginiamycin m1, sulfamerazine, and sulfamethiazole, were not detected in either watershed.

**FIG 1:**
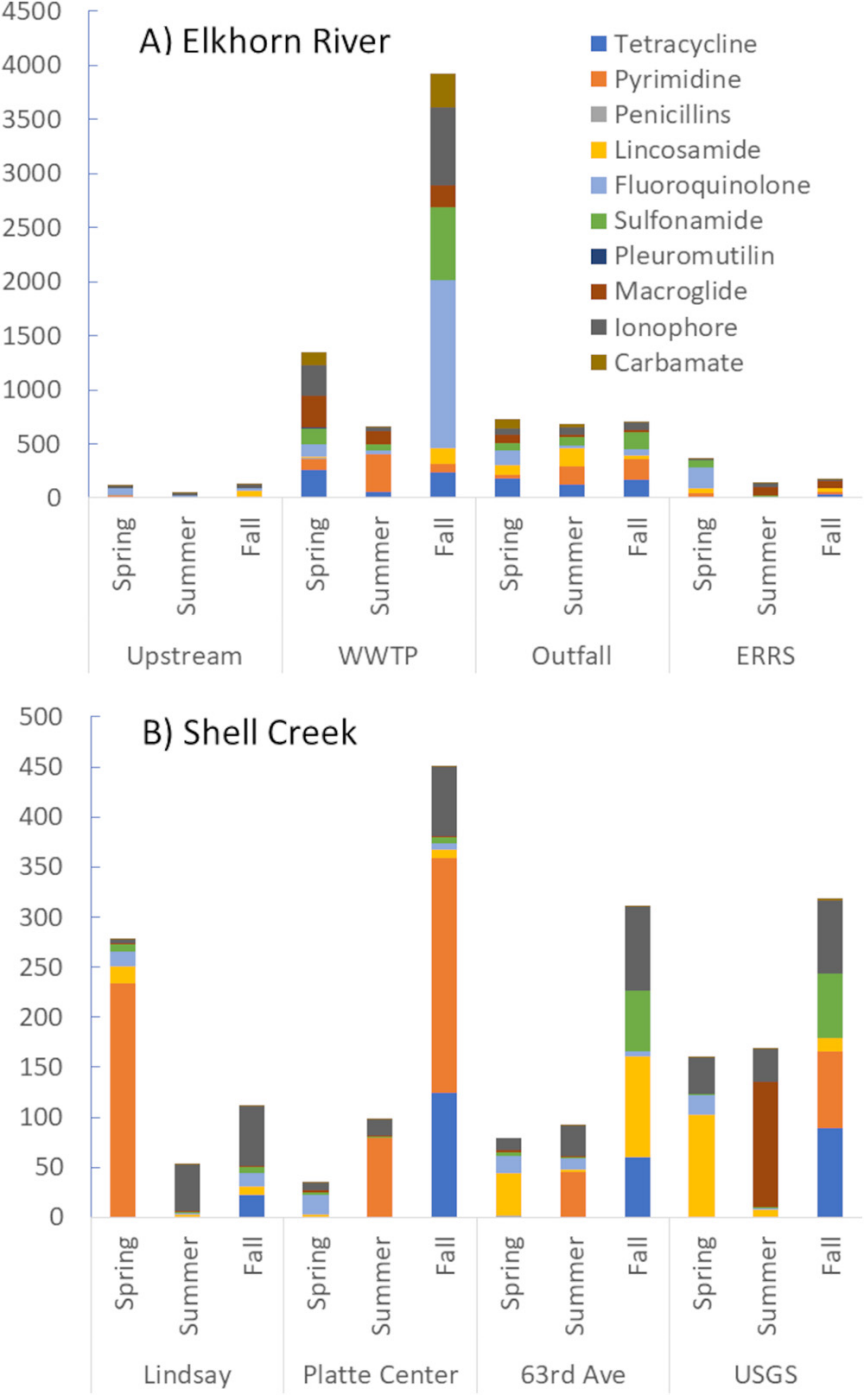
Antibiotics detected in the Shell Creek and Elkhorn River watersheds based on antibiotic class. Vertical axis are not scaled similarly.

Within the Elkhorn River watershed, total antibiotic load ranged between 46.0 and 3859.1 ng POCIS^−1^ (Fig. 1A). Total antibiotic load was significantly lower in the upstream and most downstream (ERRS) locations that were not influenced by municipal wastewater with total antibiotic concentrations at or below 370.1 ng POCIS^−1^ (Fig. 1A). In these locations, the pyrimidine antibiotic, trimethoprim, was the predominant antibiotic class detected; while the WWTP and outfall had higher levels of fluoroquinolones (26.0 – 1552 ng POCIS^−1^), ionophores (31.4 – 718.7 ng POCIS^−1^), carbamates (14.6 – 312.9 ng POCIS^−1^), macrolides (19.5 – 302.1 ng POCIS^−1^), and sulfonamides (50.3 – 680.7 ng POCIS^−1^). Total antibiotic load in Shell Creek was comparable to the upstream and downstream ERRS sampling locations on the Elkhorn River with concentrations between 45.4 and 450.2 ng POCIS^−1^ (Fig. 1B).

Total antibiotic load was highest during the fall sampling period for all sites in both watersheds except for the outfall in the Elkhorn River (Fig. 1). Fluoroquinolones were predominantly detected during the spring in both the Shell Creek and Elkhorn River watersheds with concentrations ranging between 14.5 and 195.1 ng POCIS^−1^. The Shell Creek watershed also had higher concentrations of lincosamides (16.6 – 102.0 ng POCIS^−1^) during this period (Fig. 1B); while the Elkhorn River contained higher levels of pyrimidines and tetracyclines (174.3 – 345.0 ng POCIS^−1^) (Fig. 1A). Ionophore and pyrimidine (trimethoprim) antibiotics were the predominant ones detected in summer and fall in the Shell Creek watershed with tetracyclines only detected in the fall (Fig. 1B). Seasonal trends within the Elkhorn River watershed were not as consistent as those seen in Shell Creek (Fig. 1A). In the Elkhorn River watershed, the largest difference was observed in the WWTP effluent with the highest concentrations occurring in the fall sample. This result likely corresponds with the fall sampling occurring after the end of the plant’s disinfection season, and therefore, more antibiotics would be expected in this sampling event. In Shell Creek, the fall sampling event contained the highest level of antibiotics at each sampling location. In our prior work in the Shell Creek watershed (20), we observed the highest antibiotic concentrations in the spring, however, in our prior work, we analyzed a smaller number of antibiotic classes. In the present study, we observed different antibiotic classes present at higher concentrations in the fall samples. Notably, tetracyclines and pyrimidine antibiotics contributed more (as a percentage of total antibiotic present) in the fall samples. We evaluated the discharge records for Shell Creek at the USGS gauging station and observed the lowest discharge occurring in the fall sampling window. Therefore, precipitation events carrying antibiotics from fields with land applied manures could also result in higher instream concentrations during this period.

Our sampling found six antibiotics of human significance: trimethoprim, sulfamethoxazole, tetracycline, fluoroquinolones, sulfadiazine, and erythromycin. Sulfamethoxazole and tetracycline were found predominantly associated with the WWTP and outfall in the Elkhorn River watershed with concentrations ranging between 0.2 to 65.3 ng POCIS^−1^ (Table S3). Compounds in the fluoroquinolone family were frequently detected in both watersheds with concentrations ranging between 1.86 to 19.34 ng POCIS^−1^ in the Shell Creek watershed and between 6.90 to 195.12 ng POCIS^−1^ in the upstream and ERRS sampling locations with higher concentrations measured in the WWTP and outfall (26.04 to 1551.5 ng POCIS^−1^). In 2018, the College of Veterinary Medicine released a Five-Year Plan to Support Antimicrobial Stewardship to the United States Food and Drug Administration (FDA) (21). One of the hallmarks of this recommendation was that antibiotics medically important to human use should be limited to animal usage only when necessary for treatment (21 CFR 558). Each of these antibiotics has important influence on human infection therapy, for instance, sulfa compounds are used in treatment of urinary tract infections and have induced higher incidences of ESBL resistance. Of interest was that these antibiotics of human significance were found in agricultural watersheds up to 2 years after the 2018 recommendation to FDA was forwarded.

### Microbial occurrence.

A total of 211 bacterial colonies were identified during this study (Table S4). The number of organisms identified in each watershed per season ranged between 31 to 39. Bacillus cereus and Bacillus pumilus were the most frequently detected organisms found in 70.8% (*n* = 17) of samples. Bacillus megaterium and Aeromonas veronii were detected in 58.3% (*n* = 14) and 41.7% (*n* = 10) of samples, respectively. While most *Bacillus* species are not typically of concern to human health, B. cereus can cause food poisoning when toxin-producing strains are ingested in food. B. cereus is also widely distributed in the environment and considered a normal finding in the water samples. Of the organisms identified in this study, the bacteria of concern to human health included *Aeromonas* species, Alcaligenes faecalis, Klebsiella species, Enterobacter cloacae complex, E. coli, Proteus vulgaris, Pseudomonas fluorescens*-putida* group, and Serratia marcescens.

### Phenotypic resistance profile.

Of the 211 isolates identified via MALDI-TOF MS, 37 bacteria known to cause human infections were selected for further analysis, including antimicrobial susceptibility testing. An antibiogram for each watershed was created to show the compilation of the susceptibility rate for each of the bacteria-antibiotic combinations tested (Tables 1 and 2). Each antibiogram table lists the bacteria identification, the total number of times it was tested, and the proportion susceptible to each antibiotic unless otherwise noted. If an “R” is listed, the bacterial isolate is inherently resistant to that antimicrobial agent. If a “- “is listed, the antibiotic is not normally used for treatment in human infections for that bacteria and AST was not performed.

**TABLE 1:** Elkhorn River Gram-negative bacteria antibiogram detailing the number of isolates (number of times a bacteria-antibiotic combination was tested) and the proportion of isolates susceptible to the antibiotic listed^*a*^

Organism	Number of isolates	Penicillins (beta-lactam)			Mono-bactam (beta-lactam)	Aminoglycosides			Fluoro-quinolones	Carbapenems (beta-lactam)		Cephal osporins (beta-lactam)					Trimethoprim/sulfonamide
		Ampicillin	Amp-Sulbactam	Pip/tazo	Aztreonam	Amikacin	Tobramycin	Gentamicin	Levofloxacin	Meropenem	Ertapenem	Cefepime	Ceftazidime	Cefazolin	Ceftriaxone	Cefuroxime	Trimethoprim-Sulfa
Aeromonas caviae *complex*	2	R	-	100	50	100	R	100	50	100	-	50	-	R	-	R	-
Aeromonas hydrophila	1	R	-	100	100	100	R		100	100	-	100	-	R	100	R	100
Aeromonas veronii *complex*	2	R	-	100	100	100	R	100	100	100	-	100	-	R	-	R	-
Alcaligenes faecalis	1	R	-	100	0	100	100	100	100	100	-	100	-	R	R	R	-
Enterobacter cloacae *complex*	2	R	R	100	100	100	100	100	100	100	100	100	-	R	100	R	100
Escherichia coli	1	100	100	100	100	100	100	100	100	100	100	100	100	100	100	100	100
Gram-negative rods, lactose nonfermenting	2	-	-	100	100	100	100	100	100	100	-	100	-	-	R	-	-
Gram-negative rods lactose nonfermenting, oxidase positive	4	-	-	75	75	100	100	100	100	100	-	100	-	-	R	100	-
Klebsiella oxytoca	1	R	100	100	100	100	100	100	100	100	100	100	100	R	100	100	100
Klebsiella pneumoniae	1	R	100	100	100	100	100	100	100	100	100	100	100	100	100	100	100
Proteus vulgaris	1	R	100	100	100	100	100	100	100	100	100	100	-	R	100	R	100
Pseudomonas fluorescens*-putid* a group	1	R	R	100	100	100	100	100	100	100	-	100	100	R	R	R	R
Serratia marcescens	2	R	R	50	0	100	50	100	100	100	100	100	0	R	0	R	100

a R, indicates the bacterial isolate is inherently resistant to that antimicrobial agent. -, indicates the antibiotic is not normally used for treatment in human infections for that bacteria and AST was not performed.

**TABLE 2:** Shell Creek Gram-negative bacteria antibiogram detailing the number of isolates (number of times a bacteria-antibiotic combination was tested) and the proportion of isolates susceptible to the antibiotic listed^*a*^

Organism	Number of isolates	Penicillins (beta-lactam)			Mono-bactam (beta-lactam)	Aminoglycosides			Fluoro-quinolones	Carbapenems (Beta-Iactam)		Cephal osporins (beta-lactam)					Trimethoprim/sulfonamide
		Ampicillin	Amp-Sulbactam	Pip/tazo	Aztreonam	Amikacin	Tobramycin	Gentamicin	Levofloxacin	Meropenem	Ertapenem	Cefepime	Ceftazidime	Cefazolin	Ceftriaxone	Cefuroxime	Trimethoprim-Sulfa
Aeromonas caviae complex	6	R	-	100	100	100	R	100	100	100	-	100	-	R	-	R	-
Aeromonas hydrophila	2	R	-	100	100	100	R	100	100	100	-	100	-	R	100	R	100
Aeromonas veronii complex	2	R	-	100	100	100	R	100	100	100	-	100	-	R	-	R	-
Enterobacter cloacae complex	1	R	R	100	100	100	100	100	100	100	100	100	-	R	100	R	100
Klebsiella pneumoniae	2	R	100	100	100	100	100	100	100	100	100	100	100	100	100	100	100
Pantoea agglomerans group	1	100	100	100	100	100	100	100	100	100	100	100	-	100	100	100	100
Pseudomonas fluorescens*-putid* a group	2	R	R	100	50	100	100	100	100	100	-	100	100	R	R	R	R

a R, indicates the bacterial isolate is inherently resistant to that antimicrobial agent. -, indicates the antibiotic is not normally used for treatment in human infections for that bacteria and AST was not performed.

None of the bacteria isolated from the watershed were confirmed as ESBL-producing Gram-negative rods. Most of the watershed bacteria followed predictable patterns of susceptibility normally seen when tested against human-pathogenic bacteria of the same genus and species. Local hospital antibiograms (data not shown) and clinical knowledge were used for this comparison. The S. marcescens isolates (*n* = 2) from Elkhorn River exhibited decreased susceptibility to piperacillin-tazobactam (50%), aztreonam (0%), ceftazidime (0%), and ceftriaxone (0%) (Table 1). In addition, the Gram-negative, lactose nonfermenting, oxidase positive isolates (*n* = 4) had decreased susceptibility to piperacillin-tazobactam (75%) and aztreonam (75%). The wastewater bacteria decreased susceptibility indicates the isolates from the wastewater appears more resistant than the local clinical isolates of the same genus and species (i.e., the wastewater isolates have a higher MIC than the clinical isolates). All of the Shell Creek water isolates exhibited comparable patterns of susceptibility to human-pathogenic bacteria of the same genus and species (Table 2).

### Whole-genome sequencing.

The whole genome of 16 isolates obtained from the Elkhorn River watershed were sequenced. The bacteria obtained from the WWTP were isolated in all months (April (spring), July (summer) and October (fall)) and were identified as Klebsiella oxytoca, Aeromonas caviae, A. hydrophila, Serratia marcescens, *and* Proteus sp. (Table 3). Isolates from the upstream and ERRS site were only obtained for the July sampling event and included K. pneumoniae, A. hydrophila, and Alcaligenes faecalis (Table 3). Isolates from the outfall site were obtained in July and October and included *A. veronii*, P. mendocina, Enterobacter asburiae and S. marcescens (Table 3).

**TABLE 3:** List of isolates collected from the Elkhorn River watershed, including horizontally transferred ARG and the NCBI GenBank bacterial matches^*a*^

Site	Date	Isolate species	Horizontally transferred ARG	NCBI match
Upstream	July	Klebsiella pneumonia	Beta-Lactam: *bla_SHV-26_, bla_SHV-78_, bla_SHV-98_, bla_SHV-179_, bla_SHV-194_, bla_SHV-199_*	Escherichia coli and Klebsiella pneumoniae
			**Fosfomycin:** *fos*(A)	Klebsiella pneumoniae
			**Quinolone:** *oqx*(A), *oqx*(B)	Escherichia coli
Upstream	July	Aeromonas hydrophila	Beta-Lactam: *amp*(S), *cphA7*	Aeromonas sobria and *Areromonas jandaei*	
WWTP	April	Klebsiella oxytoca	Beta-Lactam: *bla_OXY-2-7_*	Klebsiella oxytoca
WWTP	April	Aeromonas caviae	Beta-Lactam: *bla_LEN13_*	Klebsiella pneumoniae
			**Fosfomycin:** *fos*(A)	Klebsiella pneumoniae
			**Quinolone:** *oqx*(A), *oqx*(B)	Escherichia coli
WWTP	July	Aeromonas hydrophila	**Aminoglycoside:** *aac6, ant2*	Pseudomonas aeruginosa and uncultured
			Beta-Lactam: *bla_MOX-6_, bla_VEB-1_*	Aeromonas punctata and *Achromobacter xylosidans*
			**Quinolone:** *aac6, qnrVC4*	Klebsiella pneumoniae
			**Phenicol:** *cmlA1*	Pseudomonas aeruginosa
			Trimethoprim: *drfA14*	Escherichia coli
WWTP	October	Serratia marcescens	**Aminoglycoside:** *aac6*	*Serratia mascescens*
			Beta-Lactam: *bla_SRT-_*_2_	*Serratia mascescens*
			Tetracycline: *tet41*	*Serratia mascescens*
WWTP	October	Proteus *sp*	Beta-Lactam: *hug*(A)	Proteus penneri
			**Tetracycline:** *tet*(H)	Pasteurella multocida
WWTP	October	Aeromonas hydrophila	Beta-Lactam: *amp*(H), *cphA2*	*Aeromonas hydrohpila* and *Serratia marsescens*
WWTP	October	Aeromonas hydrophila	Beta-Lactam: *amp*(H), *cphA2*	*Aeromonas hydrohpila* and *Serratia marsescens*
			Tetracycline: *tet*(E)	Aeromonas salmonicida
Outfall	July	Aeromonas veronii	Beta-Lactam: *amp*(H), *imi*(H)	Aeromonas hydrophila
Outfall	July	Aeromonas veronii	Beta-Lactam: *amp*(S), *cphA7*	Aeromonas sobria *and* Aeromonas jandaei
Outfall	July	Aeromonas veronii	Beta-Lactam: *amp*(S), *bla_CEPH-A3_*	Aeromonas sobria *and* Aeromonas jandaei
Outfall	July	Pseudomonas mendocina	None	None
Outfall	October	Enterobacter asburiae	Beta-Lactam: *bla_ACT-6_, bla_ACT-10_*	Enterobacter mori
			**Fosfomycin:** *fos*(A)	Klebsiella pneumoniae and *Serratia marsescens*
Outfall	October	Serratia marcescens	**Aminoglycoside:** *aac6*	*Serratia mascescens*
			Beta-Lactam: *bla_SRT-1_, bla_SRT-2_*	*Serratia mascescens*
			Macrolide: *lnu*(C)	*Serratia agalactiae*
ERRS	July	Alcaligenes faecalis	None	None

a Antibiotics in bold represent antibiotic resistance that is not intrinsic for the isolate genera.

Whole genome sequencing was conducted on 15 isolates from the Shell Creek watershed. Isolates were obtained from the Shell Creek site on each sampling event and included isolates identified as *A. salmonicida*, A. hydrophila, *A. media*, and Acinetobacter johnsonii (Table 4). Isolates obtained during the July and October sampling events from the USGS site included *P. monteilii*, *A. veronii*, and A. hydrophila (Table 4). During July and October, A. hydrophila, *A. veronii*, and Enterobacter ludwigii were also obtained from the site on 63^rd^ St. (Table 4).

**TABLE 4:** List of isolates collected from the Shell Creek watershed, including horizontally transferred ARG and the NCBI GenBank bacterial matches^*a*^

Site	Date	Isolate species	Horizontally transferred ARG	NCBI match
USGS	July	Klebsiella variicola	Beta-Lactam: *bla_LEN13_*	Klebsiella pneumoniae
			**Fosfomycin:** *fos*(A)	Klebsiella pneumoniae
			**Quinolone:** *oqx*(A), *oqx*(B)	Escherichia coli
USGS	July	Klebsiella variicola	Beta-Lactam: *bla_LEN13_*	Klebsiella pneumoniae
			Fosfomycin: *fos(*A)	Klebsiella pneumoniae
			Quinolone: *oqx*(A), *oqx*(B)	Escherichia coli
USGS	July	Pseudomonas monteilii	None	None
USGS	October	Aeromonas veronii	Beta-Lactam: *bla_OXA-427_*	Enterobacter cloacae
USGS	October	Aeromonas hydrophila	Beta-Lactam: *amp*(H), *cphA2*	Aeromonas hydrophila
Platte Center	April	Aeromonas salmonicida	Beta-Lactam: *cphA1*	Aeromonas salmonicida
Platte Center	July	Aeromonas hydrophila	Beta-Lactam: *amp*(H), *cphA2*	Aeromonas hydrophila and Salmonella enterica
Platte Center	July	Aeromonas hydrophila	Beta-Lactam: *bla_ACT-6_, bla_ACT-10_*	Klebsiella pneumoniae and *Serratia marsescens*
			**Fosfomycin:** *fos*(A)	Enterobacter mori
Platte Center	October	Aeromonas media	Beta-Lactam: *bla_OXA-427_*	Enterobacter cloacae
Platte Center	October	Acinetobacter johnsonii	None	None
Platte Center	October	Aeromonas hydrophila	**Aminoglycoside:** *aph3, aph6*	Escherichia coli, Pseudomonas aeruginosa and Shigella flexneri
			**Fosfomycin:** *fos*(A)	Enterobacter mori
			**Tetracycline:** *tet*(B)	Shigella flexneri
63rd Ave	July	Aeromonas hydrophila	Beta-Lactam: *bla_ACT-_*_12_	Enterobacter cloacae
			**Fosfomycin:** *fos*(A2)	Enterobacter cloacae
63rd Ave	October	Aeromonas veronii	Beta-Lactam: *bla_OXA-427_*	Enterobacter cloacae
63rd Ave	October	Enterobacter ludwigii	Beta-Lactam: *bla_ACT-12_*	Enterobacter cloacae
			**Fosfomycin:** *fos*(A2)	Enterobacter cloacae
63rd Ave	October	Aeromonas veronii	None	None	

a Antibiotics in bold represent antibiotic resistance that is not intrinsic for the isolate genera.

### Watershed ARG prevalence.

**FIG 2:**
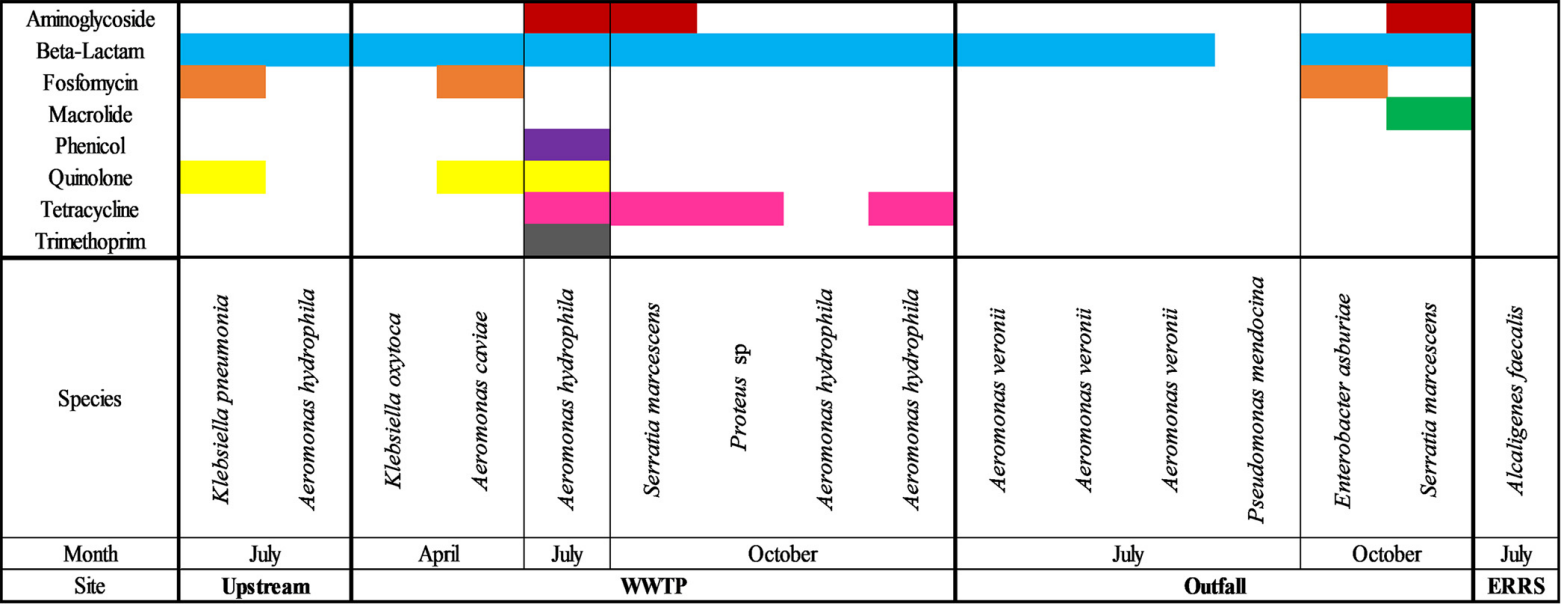
Antibiotic resistance observed in each isolate at every sampling date and site in the Elkhorn River. A colored bar denotes that resistance to that antibiotic was observed.

ARGs previously characterized as acquired by HGT were detected in 14 of the 16 isolates collected from the Elkhorn River watershed. Beta-lactam resistance genes were detected in 14 of the 16 isolates (Fig. 2). Tetracycline resistance genes were the next commonly detected ARGs (4 out of 16 isolates). They were only detected within WWTP in July and October (Fig. 2). Phenicol and trimethoprim mobile ARG were also detected in the WWTP. The detection of a greater variety of mobile ARGs in the WWTP is similar to other studies, suggesting WWTPs hosting a broad range of ARGs and MGEs (22–24).

**FIG 3:**
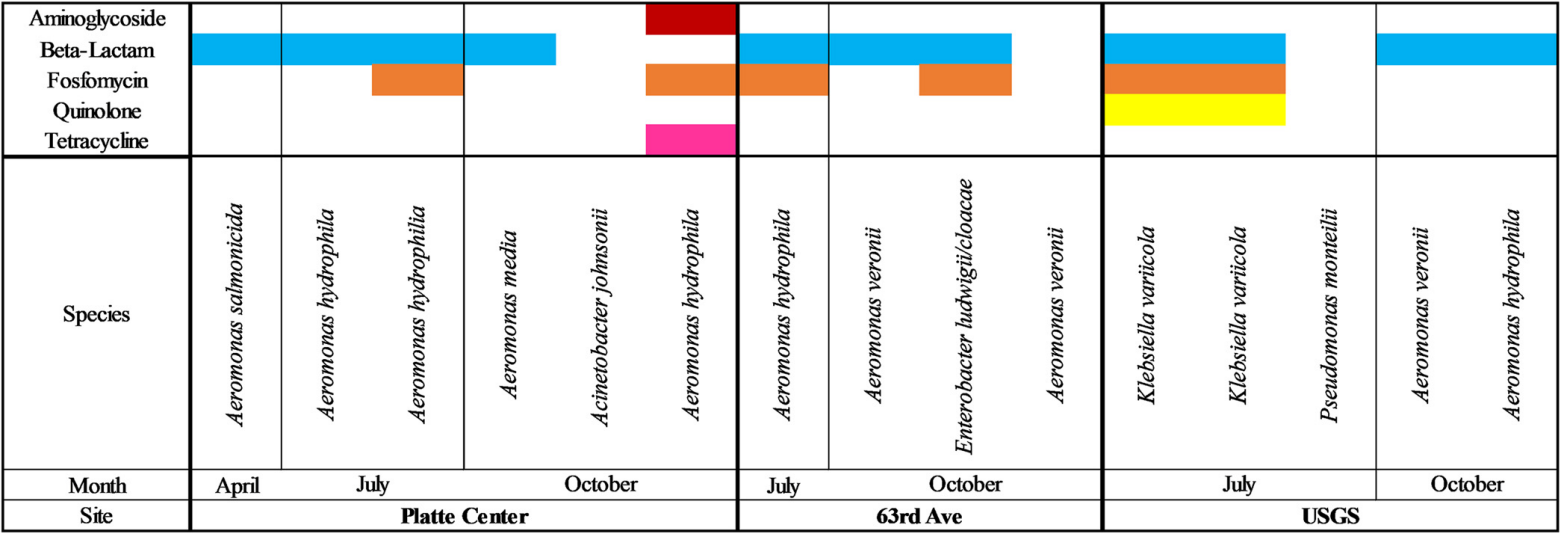
Antibiotic resistance observed in each isolate at every sampling date and site in the Shell Creek. A colored bar denotes that resistance to that antibiotic was observed.

Within Shell Creek, mobile ARGs were detected within 12 out of 15 samples. Similar to the Elkhorn River, beta-lactam ARG were the most commonly detected (11 out of 15 isolates), followed by Fosfomycin ARGs (6 out of 15 isolates), with both detected at all three sampling locations (Fig. 3).

The correlation between antibiotics and antibiotic resistance genes in the environment can be system dependent. Zhang et al. discovered correlations of some individual *tet* genes with antibiotic residues in livestock waste management systems (25), but a lack of correlation of total *tet* genes with the antibiotic residues. The systems examined here had potential for multiple inputs of antibiotics from runoff from multiple crop and animal production facilities as well as wastewater inputs for the Elkhorn River watershed. In addition, the fact that not all antibiotic transformation products, some of which could still exert antibiotic effects, could be quantified in this study may also contribute to the difficulty in establishing correlations.

### ARGs associated with HGT.

Whole genome sequencing analysis revealed that of the 16 isolates from the Elkhorn River watershed, 7 had ARGs coding for antibiotic resistance that was not intrinsic to the genus (Table 3). Genes associated with fosfomycin and quinolone resistance acquired by Klebsiella and *Aeromonas* spp. (Table 3) were most common, with neither form of antibiotic resistance intrinsic for either species (26–28). Within the Elkhorn River, aminoglycoside resistance was also acquired by A. hydrophila and S. marcescens, with A. hydrophila also acquiring resistance to tetracycline antibiotics (Table 3).

The ResFinder results from the Elkhorn River indicated that antibiotic resistance was potentially acquired from ARG previously associated with HGT from a genus different from the genus of the isolate in four cases. When assessed via the CARD and Integrall databases, seven mobile ARGs from the *A. caviae* isolated from the WWTP in April showed signs of recent HGT with Klebsiella spp. (Table 5). Further, an A. hydrophila isolated from the WWTP in July had six mobile ARGs showing recent transfer with a variety of different bacterial genera (Table 5).

**TABLE 5:** List of isolates and NCBI BLAST matches for ARG showing signs of recent HGT (>99% similarity) for the Elkhorn River

Site	Date	Isolate species	Recently transferred ARG	Drug class	Genera matched by NCBI BLAST
WWTP	April	Aeromonas caviae	*aac(6′)*	Aminoglycoside	Klebsiella
			*ompK37*	Multidrug	Klebsiella
			*kpnE*	Multidrug	Klebsiella
			*kpnF*	Multidrug	Klebsiella
			*bla_LEN10_*	Multidrug	Klebsiella
			*mar*(A)	Multidrug	Klebsiella
			*mar*(R)	Multidrug	Klebsiella
WWTP	July	Aeromonas hydrophila	*qnrVC4*	Fluoroquinolone	Klebsiella, Escherichia, Salmonella, *Citrobacter, Vibrio*
			*aac(6′)-lb9*	Aminoglycoside	Klebsiella, *Citrobacter, Vibrio,* Escherichia, *Delftia, Thauera,* Enterobacter, Pseudomonas, *Alcaligenes,* Salmonella, Acinetobacter, *Chryseobacterium, Shigella*
			*cmlA5*	Multidrug	Escherichia, Acinetobacter, *Vibrio, Yokenella, Enterobacteriaceae,* Klebsiella, Pseudomonas, *Morganella, Shewanella, Citrobacter*
			*bla_VEB-1_*	Multidrug	Klebsiella, Pseudomonas, Escherichia, *Shewanella,* Acinetobacter, Proteus, *Achromobacter, Vibrio, Serratia, Citrobacter*
			*ant(2”)-la*	Aminoglycoside	Escherichia, Enterobacter, Klebsiella, Pseudomonas, Acinetobacter, Proteus, *Citrobacter, Mannheimia, Sphingobium*
			*dfrA14*	Diaminopyrimidine	Klebsiella, Escherichia, Salmonella, Enterobacter, *Raoultella*	

Of the Shell Creek isolates, six isolates were determined to have ARG, previously characterized as being associated with HGT, for which the genera were not intrinsically resistant (Table 4). Similar to Elkhorn River, the most commonly detected ARGs associated with HGT were Fosfomycin and quinolone resistance detected in Klebsiella and *Aeromonas* spp. (Table 4). A. hydrophila also had ARGs associated with HGT for aminoglycoside and tetracycline resistance in Shell Creek (Table 4).

According to ResFinder results, four of the 15 Shell Creek isolates had ARG previously characterized as being associated with HGT from a different genus. When assessed via the CARD and Integrall databases, *Aeromonas* and Acinetobacter isolates from the Platte Center site showed evidence of recent HGT (Table 6). An A. hydrophila isolate from the July sampled had one mobile ARG showing evidence of recent HGT and the Acinetobacter isolate from the October sampling had four (Table 6). Conversely, an *A. media* isolate had two mobile ARGs and a separate A. hydrophila isolate had four mobile ARGs showing evidence of recent HGT (Table 6). The 63^rd^ street site also had an A. hydrophila isolate from July with 13 mobile ARGs showing signs of recent transfer with Enterobacter spp. (Table 6).

**TABLE 6:** List of isolates and NCBI BLAST matches for ARG showing signs of recent HGT (>99% similarity) for Shell Creek

Site	Date	Isolate species	Recently transferred ARG	Drug class	Genera matched by NCBI BLAST
Platte Center	July	Aeromonas hydrophila	*H-NS*	Multidrug	Enterobacter
Platte Center	October	Aeromonas media	*bla_MOX-9_*	Multidrug	*Citrobacter*
			*bla_OXA-427_*	Multidrug	Pseudomonas, Enterobacter, Klebsiella
Platte Center	October	Acinetobacter johnsonii	*ade*(F)	Multidrug	*Pantoea*
			*msb*(A)	Multidrug	*Pantoea*
			*knpF*	Multidrug	*Pantoea*
			*amp*(H)	Multidrug	*Pantoea*
			*emr*(R)	Multidrug	*Pantoea*
			*kpnH*	Multidrug	*Pantoea*
			*PBP3*	Multidrug	*Pantoea*
			*uhpT*	Fosfomycin	*Pantoea*
Platte Center	October	Aeromonas hydrophila	*aph(3′)-lb*	Aminoglycoside	Acinetobacter, Escherichia, *Vibrio,* Klebsiella, Salmonella, Proteus, *Alcaligenes, Citrobacter, Raoultella, Chitinibacter*
			*aph(*6*)-ld*	Aminoglycoside	Escherichia, *Citrobacter,* Salmonella, Proteus, *Vibrio, Raoultella,* Enterobacter, *Shigella*
			*tet*(B)	Multidrug	Escherichia, Salmonella, Acinetobacter, *Avibacteriuym,* Proteus, *Citrobacter, Morganella, Providencia, Neisseria, Shigella, Vibrio, Kingella, Frischella*
			*tet*(R)	Multidrug	*Glaesserella,* Escherichia, *Shigella,*Proteus, Salmonella, Acinetobacter, Klebsiella, *Avibacterium, Morganella, Vibrio*
63rd Ave	July	Aeromonas hydrophila	*amp*(H)	Multidrug	Enterobacter
			*acr*(B)	Multidrug	Enterobacter
			*acr*(A)	Multidrug	Enterobacter
			*ram*(A)	Multidrug	Enterobacter
			*oqx*(A)	Multidrug	Enterobacter
			*oqx*(B)	Multidrug	Enterobacter
			*mdf*(A)	Multidrug	Enterobacter
			*msb*(A)	Multidrug	Enterobacter
			*rlmA(II)*	Multidrug	Enterobacter
			*kpnE*	Multidrug	Enterobacter
			*kpnF*	Multidrug	Enterobacter
			*mar*(A)	Multidrug	Enterobacter
			*H-NS*	Multidrug	Enterobacter
			*mdt*(B)	Multidrug	Enterobacter
			*mdt*(C)	Multidrug	Enterobacter
			*bae*(R)	Multidrug	Enterobacter
			*acr*(D)	Multidrug	Enterobacter
			*uhpT*	Fosfomycin	Enterobacter	

Within both watersheds, aminoglycoside and tetracycline ARGs associated with HGT were detected in isolates. Both aminoglycosides and tetracyclines are commonly used in livestock operations and may reflect HGT from bacteria associated with agricultural runoff that would be prominent in both agroecosystems (29–31). Within Elkhorn River, the detection of recent HGT was exclusively found in WWTP isolates, consistent with previous studies suggesting WWTPs as a reservoirs of ARGs and MGEs (22–24).

The data provided by ResFinder, and corroborated by querying the CARD and Integrall databases, suggests that there has been recent HGT of novel ARGs to autochthonous waterborne bacteria in both watersheds. In many cases, these mobile ARGs identified in this study, predominantly in *Aeromonas* spp, were indicative of transfer potential between autochthonous bacteria and clinically relevant bacterial genera (i.e., Klebsiella, *Vibrio*, Salmonella, *Shigella*). *Aeromonas* has been found to be ubiquitous in surface waters (32, 33). Consequently, the high transfer of mobile ARG between enteric bacteria and *Aeromonas* may represent an increased reservoir of mobile ARGs in these watersheds as a consequence of agricultural runoff/WWTP discharge.

### Potential environments of the HGT associated ARGs.

**FIG 4:**
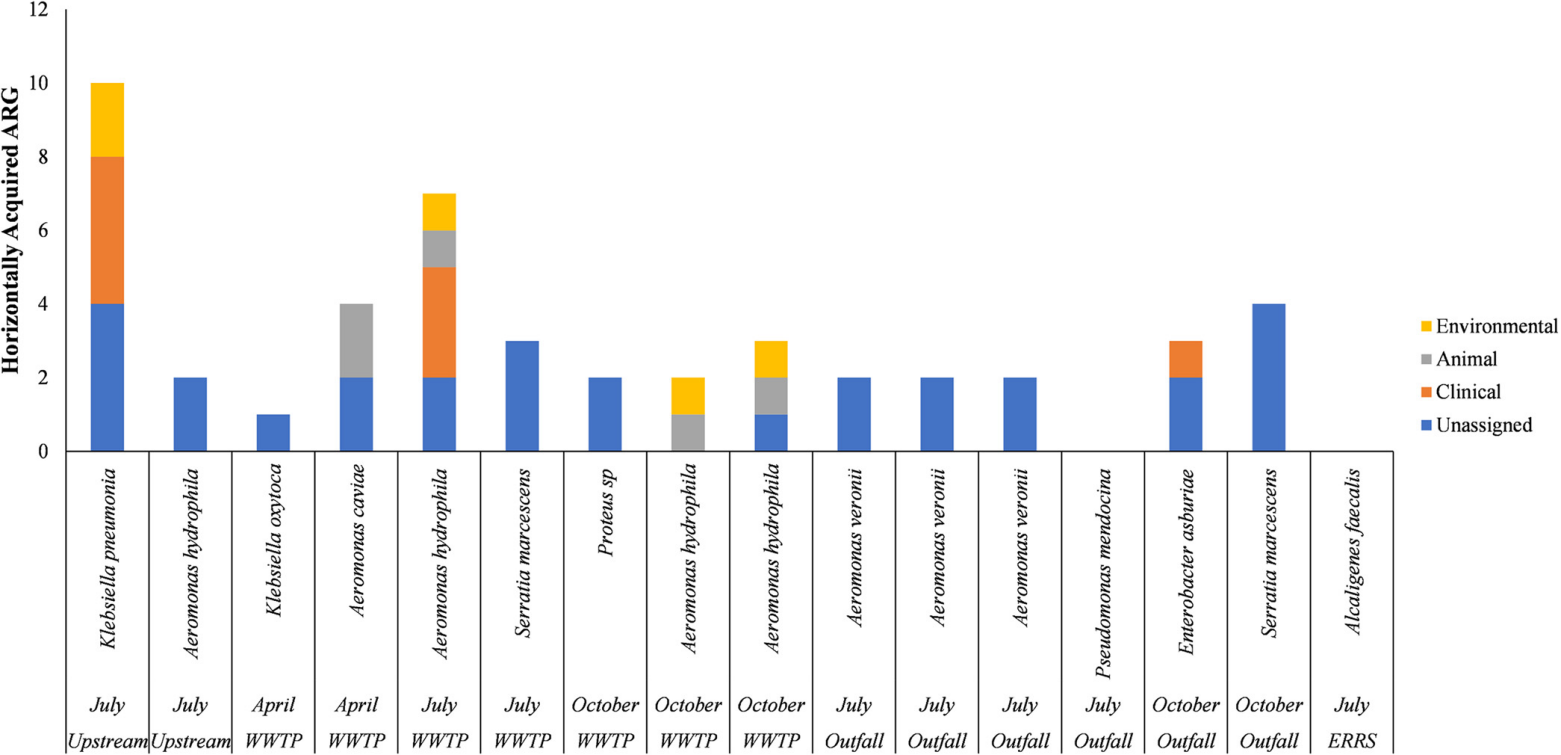
Number of mobile ARG in each isolate from the Elkhorn River matching GenBank isolates from clinical, animal, or environmental sources. Where no source was identified in GenBank, the ARG was termed “unassigned.”

**FIG 5:**
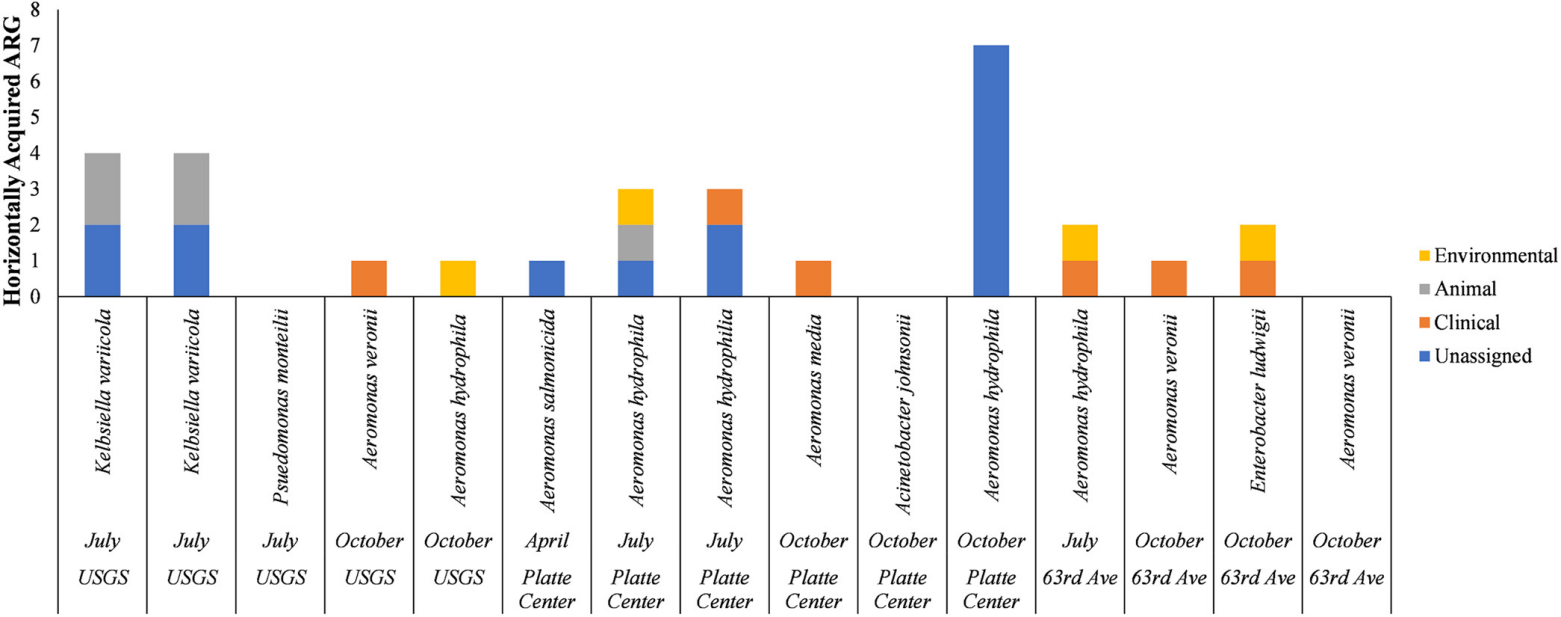
Number of mobile ARG in each isolate from Shell Creek matching GenBank isolates from clinical, animal, or environmental sources. Where no source was identified in GenBank, the ARG was termed “unassigned.”

Within both watersheds, several HGT associated ARGs had greater than 95% sequence homology with GenBank matches isolated from clinical, animal and environmental sources. Within the Elkhorn River watershed, three isolates had ARGs found in clinical matches, and four isolates ARGs found in animal and environmental bacteria (Fig. 4). Within Shell Creek, six ARG sequences had >95% homology with GenBank matches obtained from clinical isolates, while three and four isolates had matches obtained from animal and environmental sources, respectively (Fig. 5).

The amount of HGT associated ARGs in the isolates obtained from these watersheds is less than what was been reported in Hu et al. (2016). Hu et al. reported that 88% of sequences obtained from aquatic isolates had ARG matching isolates obtained from clinical or animal sources, further suggesting that ARG transfer between animal and aquatic environments occurred with the second highest frequency, behind transfer between animals and humans (34).

While these results indicate potential transfers of mobile ARGs between human/animal-associated bacteria and environmental bacteria, the large amount of waterborne *Aeromonas* isolates that may have acquired ARGs is of particular concern. A recent study has linked *Aeromonas* spp. to both disease in freshwater fish, as well as human gastrointestinal disease and wound infections (33). Due to the frequent detection of integrons in *Aeromonas* spp., the genus has been proposed as a general indicator for the spread of ARGs in water (35, 36) and in fish (37). These isolates may serve as an indicator of ARG transfer and suggest a reservoir of ARG that could be transferred to animal or human pathogens and further the spread of antibiotic resistance (38).

## CONCLUSIONS

This study utilized a One Health approach to examine two Nebraska watersheds to determine the concentration of antibiotics and antibiotic resistant profiles of Gram-negative bacteria. Six antibiotics of human significance, trimethoprim, sulfamethoxazole, tetracycline, fluoroquinolones, sulfadiazine, and erythromycin, were detected during this study at concentrations ranging between 0.2 to 345.0 ng POCIS^−1^. The bacterial isolates capable of causing human infections were tested to identify mobile ARG acquired from clinical, environmental, and animal sources. The results indicated a pervasive transfer of beta-lactam ARGs in both the Elkhorn River and Shell Creek, although all isolates had intrinsic resistance to beta-lactams. However, 43.75% and 40% of isolates in the Elkhorn River and Shell Creek, respectively, did acquire ARGs conferring novel antibiotic resistance. Several of the ARGs closely matched sequences found in the clinical setting (18.75% and 40% the Elkhorn River and Shell Creek, respectively) and in animal hosts (25% and 20% the Elkhorn River and Shell Creek, respectively). ARG sequences identified in isolates of this study showed evidence of recent HGT events, determined by >99% sequence similarity, with sequences previously characterized in clinically relevant pathogens. A large amount of the isolates in this study were identified as *Aeromonas* spp., which has been proposed as a general indicator of ARG transfer within watersheds and may represent a growing aquatic reservoir of ARGs which can be readily transferred to other human and animal pathogens.

Despite this plausible connection between animal agriculture and human health, studies linking antibiotic use in animals to human pathology have been inconclusive. One of the resistance mechanisms studied in animals has been the extended-spectrum beta-lactamases (ESBLs) found in Gram-negative organisms (GNO). This resistance mechanism is especially important in infections caused by Escherichia coli, Salmonella species, and Acinetobacter GNOs. ESBLs are enzymes that mediate resistance to extended-spectrum (third generation) cephalosporins (e.g., ceftazidime, cefotaxime, and ceftriaxone) and monobactams (e.g., aztreonam). The past 2 decades have seen an increase in both infections and colonizations caused by extended-spectrum cephalosporin resistant (ESC-R) isolates and plasmid mediated AmpC (pAMPC) resistance enzymes, both traced to ESBL production. Studies have shown that animal livestock is a prolific reservoir of ESC-R and pAmpC resistance genes (39). In addition to the previously mentioned resistance genes, ESBLs are implicated in the emerging prevalence of quinolone resistance, which relies on the mechanism of DNA-Gyrase enzyme to reduce bacterial growth in GNO infections (40). The bacteria isolated in the watersheds in this study are considered a normal finding as these organisms are ubiquitous in nature. Although most of the bacteria were not multidrug resistant at the time of isolation, our study shows the bacterial isolates do possess antibiotic resistant genes on mobile genetic elements. The horizontal gene transfer of mobile genetic elements allows otherwise harmless bacteria to hand off genes that provide resistance to antibiotics, turning them into multidrug-resistant bacteria. In addition, persistence of multidrug-resistant pathogenic bacteria may occur in areas of the watershed where antibiotics are concentrated because they may get into a physiologically resistant state without any genetic changes due to increased exposure of the antibiotics used in animal agriculture and livestock production. The results presented in this study provide evidence of transfer of highly mobile ARG between environment, clinical, and animal-associated bacteria and highlight the need for a One Health perspective in assessing the spread of antibiotic resistance.

## MATERIALS AND METHODS

### Site and sampling description.

**FIG 6:**
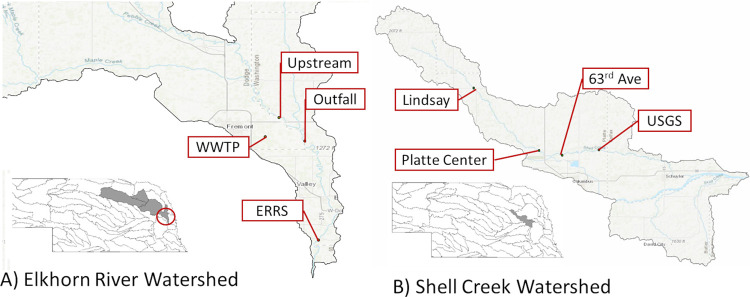
Sampling locations within A) Elkhorn River and B) Shell Creek watersheds. This map was drawn by a coauthor.

Eight sampling locations were selected within 2 watersheds, 4 in the Elkhorn River watershed and 4 in the Shell Creek watershed (Fig. 6). The Elkhorn River watershed is a large (18,100 km^2^) agriculturally impacted watershed in northeastern Nebraska. It contains a diversity of land-use applications with urban areas interspersed with both animal and row crop agriculture. Sites in this watershed were selected to include contributions from the Fremont Wastewater Treatment Plant (WWTP) which treats wastewater from both municipal and industrial sources, including food processing facilities (Fig. 6A). Shell Creek is a smaller (1200 km^2^) watershed located in east-central Nebraska (Fig. 6B). This is an intensive agricultural region with row crop production and animal agriculture with beef, swine, and poultry operations. The watershed contains over 1 million head of swine, poultry and cattle (41) and approximately 1500 people. Sampling locations within Shell Creek watershed were selected based on a previous study evaluating the occurrence of antibiotics in agricultural watersheds (20).

During 2018, the measurement of physiochemical water quality parameters was done monthly from April to October at the eight study locations. Water quality parameters, including temperature, pH, and dissolved oxygen were measured in the field (YSI Inc., Yellow Springs, OH, USA) and a 250 mL water sample collected for further analysis of nitrate, phosphate and total coliforms/E. coli. Nitrate and phosphate were quantified using colorimetric methods (HACH Company, Loveland, CO, USA); while total coliforms and E. coli were assessed using the Colilert Quanti-Tray system (IDEXX Laboratories Inc., Westbrook, ME, USA).

Polar organic chemical integrative samplers (POCIS) were used to quantify the occurrence of antibiotics and other pharmaceuticals at each of the sampling locations seasonally in the spring (April – May), summer (July – August), and fall (September – October). Triplicate POCIS (Oasis HLB, Environmental Sampling Technologies, St. Joseph, MO, USA) were loaded into stainless steel canisters (Environmental Sampling Technologies, St. Joseph, MO, USA). The canisters were submerged and secured at each sampling location using a metal chain and attached to a nearby structure or stake depending on conditions at the specific location. The POCIS were deployed for 30-day periods during each season. Upon completion of the exposure period, the POCIS were removed from the canisters and rinsed with RO water to remove dirt and debris before being wrapped individually and place on ice for transport to the lab for further processing. Coinciding with POCIS deployment, an additional 50 mL water sample was collected in plastic centrifuge tubes once during the spring, summer, and fall to evaluate the microbiological composition in each of the sampling locations.

### POCIS extraction and analysis.

Analytes were eluted from POCIS using a previously described solvent extraction (42). Briefly, the contents of each POCIS were quantitatively transferred to glass chromatography columns by rinsing with methanol. Analytes were eluted using 50 mL of dichloromethane/acetone (80:20) followed by 50 mL of methanol. Each extract was spiked with internal standards and surrogates before evaporation under a steady stream of nitrogen (RapidVap N_2_ Evaporation System, Labconco, St. Joseph, MO, USA). Antibiotic residues in concentrated extracts were quantified by three different methods using liquid chromatography with tandem mass spectrometry (LC-MS/MS).

Passive sampler extracts were analyzed by three different instrumental methods tailored to chemical properties of each group (Table S1) (42) with a third method specifically for human use compounds (Table S2). Group 1 compounds were separated and quantified using a 2695 HPLC interfaced to a Quattro Micro triple quadrupole mass spectrometer in positive ion electrospray mode (Waters Corp., Milford, MA, USA). A Thermo HyPURITY C18 column (2.1 × 250-mm, 5 μm particle size) was held at 50°C with a flow rate of 0.2 mL min^−1^. Mobile phase solvents were: A) 0.1% (vol/vol) formic acid in acetonitrile, and B) 0.1% (vol/vol) formic acid in water. Initial conditions were 5% A, hold until 1 min, then step to 50% A, hold until 3 min followed by linear gradient to reach 75% A at 14 min, then step to 100% A and hold until 20 min, then immediately back to initial conditions (0%A), hold for 8 min for total run time of 28 min. The source conditions were collision gas, Ar at 5.3 × 10^−4 ^kPa; desolvation gas, N_2_ at 700 L h^−1^; desolvation temperature, 500°C; cone gas, N_2_ at 30 L h^−1^; source temperature, 120°C; capillary, 4 kV; extractor, 3 V; and RF lens, 0.1 V.

Group 2 analytes were separated and quantified on a 1200 HPLC interfaced with a 6410 triple quadrupole operated in positive ion electrospray mode (Agilent Technologies, Santa Clara, CA, USA). A Thermo HyPURITY C18 HPLC column (2.1 × 250-mm, 5 μm particle size) was held at 50°C with a flow rate of 0.2 mL min^−1^. Mobile phase solvents were: A) 0.01% (vol/vol) formic acid in methanol, and B) 1 mM ammonium citrate in water. Initial conditions at 0% A, hold until 1.0 min followed by linear gradient to reach 80% A at 3 min and 95% A at 12 min, hold until 23 min then immediately back to initial conditions (0% A) and hold for 5 min (total run time was 28 min). Source conditions were: desolvation gas, N_2_ at 12 L min^−1^; desolvation temperature, 350°C; nebulizer, 276 kPa; capillary voltage, 4 kV; and cell accelerator voltage, 7 kV. For group 1 and 2 analytes a pseudomolecular ion [M+H]^+^ was selected as the parent ion for fragmentation, and corresponding fragment ion(s) were selected for identification and quantitation. Multiple reaction monitoring (MRM) transitions, collision energies, fragmentor and cone voltages, and retention times used for each antibiotic, internal standard, and surrogate are presented in Table S1.

Human use compounds were separated and quantified using a Waters Acquity H-class UPLC with a Premier BEH C18 1.7 μm column. The Waters Xevo-TQ-S system (Waters Corp., Milford, MA, USA) was equipped with an Unispray source operating in a positive mode. The UPLC flow rate was set to 0.6 mL/min with mobile phase consisting of solution A, 0.3% vol/vol formic acid and 0.1% wt/vol ammonium formate in water, and solution B, 1:1 methanol and acetonitrile, using the following gradient elution: Initial-1 min 100% A, 0% B;1–2 min 70% A, 30% B and hold until 3.50 min;3.50–5 min 30% A, 70% B and hold until 5.50 min; 5.50 min 0% A, 100% B and hold until 9 min;9–12 min 100% A, 0% B. MS parameters include impactor voltage 1.5kv, source temperature 150°C, desolvation temperature 600°C, Desolvation gas flow 1000 L/hr, and cone gas flow 50L/hr. The ion transitions, cone energies, collision energies, and retention times are given in Table S2. Peak integration and calculations of concentrations against the standard curve were performed using Waters Masslyxn v 4.2.

Calibration solutions for each group were made separately at internal standard concentrations of 100 pg μL^−1^ and analyte and surrogate concentrations of 0, 2, 12.5, 37.5, 62.5, and 125 pg μL^−1^. Instrument detection limits were estimated from the standard deviation of 8 replicated 25 μL injections of the lowest calibration solution, and averaged 14 ± 12 pg for group 1 compounds and 6 ± 9 pg for group 2 compounds. Laboratory reagent blanks and fortified blanks were prepared by processing equivalent solvent volumes used to extract POCIS sorbent. Recovery of laboratory fortified blanks analyzed at the same time as the passive sampler extracts averaged between 39 ± 40 for penicillin G and 108% ± 41% for group 1 and between 53.2 ± 49.1% for lincomycin and 146% ± 61% for ractopamine in group 2 compounds. Surrogate recoveries for group 1 and group 2 POCIS samples averaged 88.9% ± 40% and 38% ± 30%, respectively. Contaminant concentrations in all laboratory reagent blanks were below detection.

### Culture isolation and identification protocol.

The 50 mL water samples were transported in coolers to the University of Nebraska Medical Center (UNMC) for bacteria isolation. These were centrifuged at 2,500 rpm for 10 min to concentrate suspended solids and bacteria present. The solids fraction was inoculated onto three types of agar media for culture: Sheep’s Blood Agar (SBA) Medium, MacConkey (MAC) Agar Medium and HardyCHROM ESBL Agar (Hardy Diagnostics, Santa Maria, CA, USA). The SBA was used as a general purpose nonselective medium to grow a wide variety of Gram-positive and Gram-negative organisms. It provided the ability to visualize the different colony morphologies of the bacteria to isolate for further identification and susceptibility testing. The MAC Agar is a selective and differential medium for Gram-negative rods and was used in conjunction with the SBA for colony morphology recognition of the bacteria isolated.

Screening for the presence of ESBL producing *Enterobacteriaceae* that were potentially nonsusceptible to ceftazidime and cefpodoxime was done using HardyCHROM ESBL Agar (33). If suspicious growth was present on the selective and differential HardyCHROM ESBL agar, confirmatory identification testing and antimicrobial susceptibility testing (AST) was performed using a MicroScan WalkAway plus System (Beckman Coulter Inc., Brea, CA, USA). AST was performed using the microdilution method to determine a MICs for the detection of antimicrobial resistance, including the detection of ESBL-producing isolates. The numbers and types of isolates evaluated is presented in Table S4.

The different bacterial isolates from the three water sample cultures for all sites were sent to the Nebraska Veterinary Diagnostic Center for identification via Matrix-Assisted Laser Desorption/Ionization-Time of Flight mass spectrometry (MALDI-TOF MS) using a Bruker Microflex LT instrument with Biotyper software (Bruker Corp., Billerica, MA, USA). The identifications of the bacteria were then reviewed and evaluated by the research team. Based on professional knowledge and expertise by the research team’s physician assistant and clinical microbiologist, those organisms which are opportunistic pathogens and can cause human infections had further testing performed, including antimicrobial susceptibility testing and whole-genome sequencing. Of the bacteria identified in this study many were environmental organisms which do not commonly cause disease in humans (ex: *Bacillus* spp and *Lysinibacillus* spp) and did not warrant further evaluation. Only those opportunistic pathogenic bacteria that cause human infections were selected for further analysis.

### DNA extraction, library preparation, and sequencing.

Isolated bacterial cultures were grown overnight at 35°C in 1.5 mL tryptic soy broth (Becton Dickson) and DNA was extracted using the GenElute Bacterial Genomic DNA kit Protocol (Sigma-Aldrich) as per the manufacturer’s instructions. Library preparation for whole-genome sequencing was performed by the UNMC Genomics Core Facility. A Nextera DNA Flex Library Prep kit (Illumina, Inc. San Diego, CA) was used for library preparation and 150 bp paired-end reads were sequenced using an Illumina NextSeq 500 (Illumina, Inc. San Diego, CA) with a mid-output flow cell, at an expected sequencing depth of 1.2 Gb per sample.

### Bioinformatic analyses.

Raw reads were trimmed and filtered using Trim Galore! and FASTQC to remove low quality reads and adapter sequences (43). Filtered reads were then assembled using SPAdes with multiple Kmer values (7, 9, 13, 17, 21, 25, 29, 33, 37, 41, 45, 49, 53, 57, 61, 65, 69, 73, 77, 81, 85, 89, 93, 97, 101, 105, 109, 113, 117, 121, 125) and a phred offset of 33 (44), followed by quality assessment using QUAST (45). Information regarding the GC%, N50, total size, largest contig and number of contigs can be found in Tables S5 and S6 The assembled contigs were then run using ResFinder to identify ARG associated with acquisition via HGT (46). The ResFinder program can accurately predict resistance phenotypes from WGS data (47). Matches obtained from ResFinder had >95% similarity to isolates stored in the NCBI GenBank database (48). The accession numbers obtained from ResFinder were then queried to determine whether the source from which the bacterial matches were obtained was identified (i.e., from a clinical, agricultural, or environmental source). If no mention of where the bacterial matches was identified, the environmental source was noted to be “unassigned.”

To further assess mobile ARG, assembled contigs were annotated for ARGs using the resistance gene identifier (RGI) with default parameters against the comprehensive antibiotic resistance database (CARD) (49). Contigs containing ARGs were then further annotated using a BLAST search against the Integrall database to identify MGE-containing contigs (50, 51). Where an ARG was present on a contig containing an MGE, these ARGs were considered to be potentially mobile. A further NCBI BLAST search of the nucleotide sequence of these mobile ARG was then conducted to identify whether there had been recent HGT between genera, as determined by a >99% sequence identity (34, 52).

### Data availability.

The genomic data from this study can be accessed at GenBank under the accession number PRJNA771187.
