# Supplementary material for: The Human Health Implications of Antibiotic Resistance in Environmental Isolates from Two Nebraska Watersheds
Source: Microbiol Spectr. 2022 Mar 21;10(2):e02082-21. doi: 10.1128/spectrum.02082-21 (PMC9045274; doi:10.1128/spectrum.02082-21)
Supplement: SUPPLEMENTAL FILE 1 — Supplemental material. Download SPECTRUM02082-21_Supp_1_seq10.pdf, PDF file, 0.3 MB [file spectrum02082-21_supp_1_seq10.pdf]

Supplemental Material

Table S1. Liquid chromatography-mass spectrometry parameters for the analysis of the antibiotics, internal standards, and surrogates.

		Compound	MRM Transitions m/z		m/z	Collision energy (eV)	Cone Voltage (V)	Retention Time (Min)
Group 1	Antibiotic	Ampicillin	350	>	106	15	15	7.8
		Ceftiofur	524.5	>	241.1	15	15	8.9
		Anhydroerythromycin	716.5	>	158	30	30	9.6
		Erythromycin A	734.4	>	158	30	30	8.9
		Novobiocin	613	>	189	26	20	13.0
		Tiamulin	494.3	>	191.9	24	32	9.2
		Tylosin	916.9	>	174.2	35	50	8.9
		Virginiamycin M1	526	>	355.1	18	24	10.4
	Internal std	Penicillin V	351	>	160	14	15	10.1
		Roxithromycin	837.6	>	158	35	35	9.4
	Surrogate	Oleandomycin	688.6	>	544.1	18	25	8.7
Group 2	Antibiotic	Chlortetracycline	479	>	462	110	21	11.3
		Lincomycin	407	>	126	110	25	11.1
		Monensin (Na^+ adduct)	693	>	461	50	50	20.2
		Oxytetracycline	461	>	426	120	20	11.3
		Sulfadiazine	251	>	156	110	15	10.56
		Sulfadimethoxine	311	>	156	80	20	11.7
		Sulfamerazine	265	>	156	110	15	10.9
		Sulfamethazine	279	>	156	90	10	11.2
		Sulfamethizole	271	>	156	80	10	11.0
		Sulfamethoxazole	254	>	156	110	10	11.2
		Sulfathiazole	256	>	156	100	21	10.7
		Tetracycline	445	>	410	102	17	11.3
		Trimethoprim	291	>	230	110	20	10.9
	Internal std	Doxycycline	445	>	428	120	15	11.9
		Salinomycin	773.5	>	431	130	50	21.6
		Sulfamethazine-13C6	285	>	186	90	20	11.2
	Surrogate	Sulfachloropyridazine	285	>	156	90	5	11.2

Table S2. Liquid chromatography-mass spectrometry parameters for the analysis of human use pharmaceutical (HUS) compounds, internal standards, and surrogates.

Compound	Parent-Daughter m/z	Cone (V)	Collision (V)	Retention Time (min)
13C3,15N-Acetominophen (IS)	155.108>92.95<br>155.108>44.939	36<br>36	24<br>26	2.01
13C3,15N-Ciprofloxacin (IS)	336.068>318<br>336.068>248.106	54<br>54	20<br>24	1.70
13C3-Caffeine (IS)	198.047>140.034<br>198.047>42.926	46<br>46	28<br>46	2.53
13C3-Trimethoprim (IS)	294.14>126.042<br>294.14>264.043	64<br>64	30<br>32	2.63
13C6-Carbamazepine (IS)	243.11>171.049<br>243.11>184.905	42<br>42	50<br>44	4.71
13C6-Sulfamethazine (IS)	285.067>97.975<br>285.067>123.972	44<br>44	44<br>34	2.69
13C6-Sulfamethoxazole (IS)	260.134>98.071<br>260.134>162.002	16<br>16	38<br>20	2.87
13C6-Thiabendazole (IS)	208.062>180.982<br>208.062>137.003	54<br>54	22<br>32	2.62
13C-N-Methylerythromycin-d3 (IS)	738.398>82.986<br>738.398>162.13	16<br>16	70<br>48	5.02
Cotinine-d3 (IS)	180.134>80<br>180.134>100.949	52<br>52	32<br>30	1.08
Fluoxetine-d6 (IS)	316.17>43.964<br>316.17>154.134	34<br>34	20<br>10	5.25
13C3-Atrazine (Sur)	219.117>97.966<br>219.117>105.903	46<br>46	32<br>40	4.86
13C3-Desethylatrazine (Sur)	191>149	30	20	2.98
Orphenadrine (Sur)	270.093>181.129<br>270.093>165.258	12<br>12	16<br>62	4.91
Oleandomycin (Sur)	688.381>158.159<br>688.381>544.271	42<br>42	30<br>18	4.76
1,7-dimethylxanthine	181.2>124<br>181.2>96	40<br>40	25<br>35	2.32

Table S3. Veterinary and human use antibiotics detected in POCIS deployed in the Elkhorn River and Shell Creek watersheds presented as ng POCIS^{-1}.

	Location	Date	Ampicillin	Azithromycin	Chlortetracycline	Ciprofloxacin	Clinafloxacin	Danofloxacin	Enrofloxacin	Erythromycin	Erythromycin Anhydro-	Flumequine	Lincomycin	Lomefloxicin	Monensin	Norfloxacin	Ofloxacin	Oxytetracycline	Penicillin G	Roxithromycin	Sarafloxacin	Sulfachlorpyridazine	Sulfadiazine	Sulfadimethoxine	Sulfamethazine	Sulfamethoxazole	Sulfanilamide	Sulfathiazole	Tetracycline	Thiabendazole	Tiamulin	Trimethoprim	Tylosin
Elkhorn River	Upstream	Spring	3.2					16.0	16.4	1.5		0.3	5.3	4.5	22.5		12.5				5.2		0.1	0.0						0.8		25.8	
		Summer	1.2					6.5		0.7	0.2		1.1	0.4	22.8									0.1						0.7		12.1	0.2
		Fall	0.4	0.2	7.6			1.0			0.4		62.8		28.2		15.4							0.1	0.8	1.6		0.2		0.2		109.2	
	WWTP	Spring	2.0	13.1	245.3			7.1		8.6	145.6		19.7	4.1	276.4		90.7	2.8			4.2			2.4	16.5	134.4			7.0	119.2	1.7	345.0	134.8
		Summer	0.4	5.4	49.8			6.2			67.5		3.6	0.6	31.4		20.1	3.6			4.8			3.4		46.9			5.2	14.6	0.5	82.7	54.6
		Fall		12.2	101.4						44.7		141.1		718.7		1551.5	65.7					2.4	122.7	66.7	487.1		1.8	65.3	312.9	1.9	23.6	139.6
	Outfall	Spring		4.8	181.6			10.3		4.9	28.7		92.6	4.5	52.3		127.4	0.4						1.2	11.1	47.4			2.5	93.5	0.4	174.3	44.0
		Summer		2.0	115.5					2.7	9.7		163.6	2.8	67.2		21.2				2.0			1.2	6.2	68.5			2.4	32.1	0.1	191.1	12.8
		Fall		2.1	148.9					0.8	7.6		35.7		65.2		54.8	4.2						3.6	14.5	143.5		0.3	10.6	19.3	0.2	41.7	9.0
	ERRS	Spring	3.5	1.3		42.9		23.2	38.1	0.8		0.5	42.7	13.2	13.2	31.9	25.0				20.7				1.2	7.8	53.7			0.5			0.1
		Summer	0.2		2.4			3.4		2.3			3.3	1.7	37.6		3.0			79.1					1.7	2.8				0.8		29.1	0.3
		Fall	1.2	0.1	29.1					0.3			24.6		11.7					57.4				0.8	4.4	4.8				1.1		234.3	0.1
Shell Creek	Lindsay	Spring	0.1					3.7		0.4	1.0		16.6		3.4		9.4		0.8		1.4			4.9	1.4	0.9				1.2	0.1		0.2
		Summer	0.2	0.3						0.6			2.3	1.9	46.6									0.3	0.9					0.7			
		Fall		0.2	22.2		6.8			0.4			8.3		59.8		7.4						0.2	0.2	5.5					0.6			
	Platte Center	Spring	0.1					10.0		1.0	1.0		2.3	1.4	7.2		6.3		0.7		1.6			0.1	2.2					0.1			0.3
		Summer	0.8	0.7											15.6											1.6				1.0		78.9	
		Fall			124.1								8.7		69.2		5.0							1.8	5.3					0.6		235.2	0.3
	63rd Ave	Spring	1.8					7.6		0.2	2.1		42.0	1.6	12.0						7.7			1.6	1.4	1.1							0.4
		Summer	0.1	0.5					8.1				2.4		30.9		2.5					0.3			1.1					1.1		45.9	0.2
		Fall	0.5		59.9						0.3		99.9		83.2		6.0							14.3	45.5					1.1			0.3
	USGS	Spring	0.3				0.9	6.7		0.1			102.0	5.7	36.7		5.3				0.7				1.0	0.3				0.1			0.2
		Summer	0.4	0.2									7.1		33.1		1.9			124.2				0.2	1.0					0.9			0.2
		Fall			88.6			4.3			0.3		14.3		73.2									11.7	51.9					2.0		76.6	0.2

Table S4. Results of organism identification.

	Shell Creek												Elkhorn River											
	Spring				Summer				Fall				Spring				Summer				Fall			
**Isolate Identification**	Lindsay	Platte Center	63rd Ave	USGS	Lindsay	Platte Center	63rd Ave	USGS	Lindsay	Platte Center	63rd Ave	USGS	Upstream	WWTP	Outfall	ERRS	Upstream	WWTP	Outfall	ERRS	Upstream	WWTP	Outfall	ERRS
Acinetobacter johnsonii										1														
Aerococcus viridians										1														
Aeromonas bestarium																							1	
Aeromonas caviae						1				1				1										2
Aeromonas eucrenophila																							1	
Aeromonas hydrophila						2	1			1		1					1	1				2		1
Aeromonas jandaei																						1		
Aeromonas media									1		1	1												
Aeromonas salmonicida		1		1																				
Aeromonas veronii					1	2	1	1			2	1		1			1	1	4					
Alcaligenes faecalis																				1				
Anthrobacter cumminsii		1																						
Anthrobacter sp.		1																						
Bacillus altudinis									1		1	1												
Bacillus arsenicus													1											
Bacillus cereus	1		1			1	2	1	4	3	2	2		1			1	2		1	3	1	2	2
Bacillus clausii															1									
Bacillus indicus								1					1			1								1
Bacillus licheniformis			1	2				1									1	2						
Bacillus marisflavi																		1					1	
Bacillus megaterium	2	2	1		1	1	1		1				2		2	2	1			1			1	1
Bacillus muralis									1															
Bacillus niacini									1															
Bacillus pseudomycoides																				1				1
Bacillus pumilus		1	2	1		1	2	3	2		3	1			1	1	2		1	3	2	1	1	
Bacillus simplex									1															
Bacillus sp.	3	3	3	4					1				2	1	3	3					1			2
Bacillus subtilis					1		1						1						1					
Bacillus thuringiensis																				1				
Brevibacillus laterosporus																1								
Enterobacter asburiae																							1	
Enterobacter cloacae								1			1									1				
Enterobacter ludwigii											1													
Escherichia coli																	1							
Fictibacillus arsenicus																					1		1	
Klebciella variicola								2																
Klebsiella oxytoca														1										
Klebsiella pneumonia																	1							
Lactococcus lactis					1																			
Lelliottia amnigena																					1			
Lysinibacillus boronitolerans														1				1						1
Lysinibacillus fusiformis							1																	
Lysinibacillus sp																						1		
Lysinibacillus sphaericus				1																				
Plesiomonas shigelloides																			1	1				
Proteus sp.																						1		1
Pseudomonas mendocina																			1					
Pseudomonas monteilii								1																
Pseudomonas sp.	1																							
Serratia marcescens																						1	1	
Staphylococcus sciuri										1											1			
Streptococcus henryi														1										
Unknown organisms	1	3		1								1	3	2	3	2						1		

Table S5. Values for each Elkhorn River isolate including GC%, N50, total size, largest contig and number of contigs.

Month	Site	Isolate	GC%	N50	Total Size (bp)	Largest Contig (bp)	# Contigs
April	WWTP	*Klebsiella oxytoca*	54.9	195070	6156433	470991	87
	WWTP	*Aeromonas caviae*	57.2	544441	5751067	1719865	24
July	Upstream	*Klebsiella pneumonia*	57.68	2904416	5062271	2904416	12
	Upstream	*Aeromonas hydrophila*	58.59	279328	4597047	611747	36
	WWTP	*Aeromonas hydrophila*	61.37	65692	4503029	236927	148
	Outfall	*Aeromonas veronii*	61.12	451127	4737447	975817	28
	Outfall	*Aeromonas veronii*	58.64	144025	4604018	453403	79
	Outfall	*Aeromonas veronii*	58.86	272145	4434879	782697	27
	Outfall	*Pseudomonas mendocina*	63.45	438185	8486636	1728585	2089
	ERRS	*Alcaligenes faecalis*	56.79	1029599	4103697	1160345	14
	WWTP	*Serratia marcescens*	59.19	101191	5019417	296538	105
October	WWTP	*Proteus sp*	37.75	2059293	3918516	2059293	14
	WWTP	*Aeromonas hydrophila*	60.99	258805	4910983	1345541	41
	WWTP	*Aeromonas hydrophila*	61.56	130254	4830323	335082	77
	Outfall	*Enterobacter asburiae*	55.56	258835	4855237	903953	64
	Outfall	*Serratia marcescens*	53.88	418995	6977150	1698103	44

Table S6. Values for each Shell Creek isolate including GC%, N50, total size, largest contig and number of contigs.

Month	Site	Isolate	GC%	N50	Total Size (bp)	Largest Contig (bp)	# Contigs
April	Platte Center	*Aeromonas salmonicida*	58.51	1045828	4776132	1639596	20
July	USGS	*Kelbsiella variicola*	57.2	544919	5751067	1719335	22
	USGS	*Kelbsiella variicola*	56.92	478123	6060927	1129126	38
	USGS	*Pseudomonas monteilii*	53.96	176735	10228960	966499	189
	Platte Center	*Aeromonas hydrophila*	61.38	375834	4743596	1884174	27
	Platte Center	*Aeromonas hydrophilia*	47.5	63879	8561056	1029899	2520
	63rd Ave	*Aeromonas hydrophila*	54.58	2756786	4996783	2756786	22
October	USGS	*Aeromonas veronii*	61.48	744034	4468997	2105222	29
	USGS	*Aeromonas hydrophila*	61.49	367629	4715519	498400	37
	Platte Center	*Aeromonas media*	61.32	151447	4425210	303041	67
	Platte Center	*Acinetobacter johnsonii*	54.32	574971	4616249	2213893	13
	Platte Center	*Aeromonas hydrophila*	55.46	203237	9374710	1207270	93
	63rd Ave	*Aeromonas veronii*	61.19	363147	4473388	741149	34
	63rd Ave	*Enterobacter ludwigii*	54.48	321709	4963777	1227066	30
	63rd Ave	*Aeromonas veronii*	45.98	610642	4575474	1160860	311
